# Effect of pantoprazole and its interactions with vecuronium on the neuromuscular junction

**DOI:** 10.4103/0253-7613.62410

**Published:** 2010-02

**Authors:** Tejas K. Patel, Parvati B. Patel, C.B. Tripathi

**Affiliations:** Department of Pharmacology, Government Medical College, Bhavnagar, Gujarat, India

**Keywords:** Drug interaction, neuromuscular junction, pantoprazole, vecuronium

## Abstract

**Objective:**

To study the effect of pantoprazole on neuromuscular transmission and its interactions with vecuronium at the neuromuscular junction (NMJ).

**Materials and Methods:**

Effect of pantoprazole on neuromuscular transmission (2 μM – 16 mM) and reversal of neuromuscular blockade by pantoprazole and vecuronium with neostigmine (3.3 μM), 3,4-diaminopyridine (0.25 mM), KCl (6 mM), and CaCl_2_ (10 mM) were studied by the indirect and direct stimulated preparation of rat phrenic nerve hemidiaphragm. Cumulative reponse curves (CRC) of vecuronium (1 μM to 32 μM) were studied in the absence and presence of 32 μM, 64 μM, and 128 μM pantoprazole. Time for head drop by vecuronium infusion was recorded in the absence and presence of acute and chronic administration of pantoprazole (1.9 mg/kg) in rabbits.

**Results:**

Pantoprazole potentiated the basal contractile twitch responses at a lower concentration followed by neuromuscular blockade at a higher concentration. The neuromuscular blockade was not reversed by neostigmine (3.3 μM), 3,4-diaminopyridine (0.25 mM), KCl (6 mM), and CaCl_2_ (10 mM). Pantoprazole potentiated the vecuronium-induced neuromuscular blockade. It decreased the total time for complete blockade in rat phrenic nerve hemidiaphragm preparation (*P* < 0.05) and decreased the time for the head drop in rabbits with vecuronium infusion (*P* < 0.0001).

**Conclusion:**

Pantoprazole has a direct neuromuscular blocking action. It has the potential to interact with vecuronium.

## Introduction

Proton pump inhibitors (PPIs) are widely used in modern medicine. They are the most effective antisecretory drugs available for controlling gastric acid acidity and volume in the treatment of moderate-to-severe gastroesophageal reflux disease, a hypersecretory state such as the Zollinger-Ellison syndrome, peptic ulcers, NSAID-induced ulcers, and eradication of *Helicobacter pylori* (*H. pylori*) with two antibiotics.[1–3] Pantoprazole provides earlier healing and superior pain relief in peptic ulcer and gastroesophageal reflux disease as compared to omeprazole and H_2_ receptor antagonists.[4,5] Aspiration pneumonitis is a recognized postoperative complication of anesthesia, which is associated with high morbidity and mortality.[6,7] It is caused by aspiration of gastric contents, of pH below 2.5, with a volume exceeding 0.4 ml/kg.[8] PPIs are an effective preanesthetic medication to reduce gastric acid secretion and to prevent aspiration pneumonitis intra- and postoperatively.[9] Intravenous pantoprazole, administered one hour before surgery, is useful in decreasing the volume and increasing the pH of gastric contents, thus reducing the proportion of patients at risk of significant lung injury.[10–12]

Neuromuscular blocking drugs are agents that promote skeletal muscle relaxation and are administered routinely for tracheal intubation, during abdominal surgery, for orthopedic manipulation, and brief procedures such as laryngoscopy, bronchoscopy, and esophagoscopy. Effects of H_2_-receptor antagonists, cimetidine, ranitidine, and roxatidine have been documented on neuromuscular transmission. Cimetidine potentiates the neuromuscular action of gallamine[13] and succinylcholine[14] *in vivo*, in rats. In contrast, ranitidine reverses the action of gallamine,[15] but produces an effect similar to cimetidine against succinylcholine.[16] Roxatidine delays the onset and decreases the total time for the compete block of vecuronium in rats.[17] Famotidine has been shown to have a negligible effect on neuromuscular transmission.[18] PPIs are substituted benzimidazoles that resemble H_2_-receptor antagonists in structure, but have a completely different mechanism for antisecretory action.[19] In case of PPIs, there are documented effects of omeprazole[20] and lansoprazole[21] on neuromuscular transmission, but limited information is available on the actual mechanism. No such information is available for the effect of pantoprazole on neuromuscular transmission. In patients who receive PPIs as a premedication and chronically for the treatment of peptic ulcer, there is a potential for interaction with the neuromuscular blocking drugs. This interaction would be clinically relevant because of respiratory depression and prolonged apnea.

The present study is designed to elucidate the mechanism of action of pantoprazole on neuromuscular transmission and its potential for interaction with the competitive neuromuscular blocker, vecuronium, using the isolated phrenic nerve hemidiaphragm preparation of rat and rabbit head drop method.

## Materials and Methods

All the experiments were performed after prior permission from the Institutional Animal Ethics Committee, Government Medical College, Bhavnagar (Gujarat).

**Experimental animals:** Male albino rats of Wistar strain (200 to 300 gms) and New Zealand white rabbits (1 – 1.5 kg) of either sex were procured from the central animal house of the institute. They were housed in standard polypropylene and stainless steel cages, respectively, under controlled room temperature (24 ± 2°C) and relative humidity (60 – 70%) in a 12 hour light–dark cycle. The rats were given the standard laboratory diet and water *ad libitum*. Food was withdrawn 12 hours before the experiments.

**Drugs used:** The following drugs were used in the study: pantoprazole sodium (Mankind Pharma Ltd., New Delhi, India), vecuronium bromide (Neon Laboratories Ltd., Mumbai, India), neostigmine methylsulfate (Hindustan Pharmaceuticals, India), 3,4-diaminopyridine (Alfa Aesar, Lancaster).

Pantoprazole sodium and vecuronium powder were dissolved in normal saline and distilled water, respectively, and appropriate dilutions were made in the physiological salt solution. Neostigmine ampules (0.5 mg/ml) were used as such. 3,4-diaminopyridine was dissolved in distilled water. Stock solutions were stored in the refrigerator and dilutions were made from the stock solutions, as required, immediately before the study commenced.

**Isolated rat phrenic nerve hemidiaphragm preparation:** After sacrificing the rat, the phrenic nerve diaphragm was dissected out by opening the thoracic and abdominal wall, just below the diaphragm, on the left side, and mounted as described by Bulbring.[22] The preparation was set up in an organ bath containing 40 ml of tyrode with double glucose salt solution of the following composition (mM/liter): NaCl 136.9, KCl 2.68, CaCl_2_ 1.36, MgCl_2_ 0.98, NaH_2_PO_4_ 0.32, and glucose 11.1. It was maintained at 37 ± 0.5°C and aerated with oxygen.

Each preparation was allowed to equilibrate for 45 – 60 minutes under the resting tension of 1 – 2 g, to obtain the stable basal conditions. The preparation was stimulated indirectly (2 V) through the phrenic nerve and directly (20 V) with square wave pulses of 0.5 msec, for a duration of 0.5 Hz with a physiograph stimulator. For direct stimulation only the diaphragm was mounted and the resulting isometric muscle contractions were quantified with a forced displacement transducer and recorded on a student physiograph (INCO Biodevice, Ambala) through a strain gauge coupler. The contact time for each drug was five minutes. Six preparations were used for each set of experiments. After stabilization, the following sets of experiments were performed:

### Effect of pantoprazole on neuromuscular transmission:

**Table:** 

Group 1:	Cumulative response curve (CRC) of pantoprazole (2 *μ*M to 16 mM) in the indirectly stimulated preparation.
Group 2:	CRC of pantoprazole (2 *μ*M to 16 mM) in the directly stimulated preparation.

### Interaction between pantoprazole and vecuronium:

**Table:** 

Group 3:	CRC of vecuronium (1 *μ*M to 32 *μ*M) in the absence of pantoprazole in the indirectly stimulated preparation.
Group 4:	CRC of vecuronium (1 *μ*M to 32 *μ*M) in the presence of 32 *μ*M pantoprazole in the indirectly stimulated preparation.
Group 5:	CRC of vecuronium (1 *μ*M to 32 *μ*M) in the presence of 64 *μ*M pantoprazole in the indirectly stimulated preparation.
Group 6:	CRC of vecuronium (1 *μ*M to 32 *μ*M) in the presence of 128 *μ*M pantoprazole in the indirectly stimulated preparation.

The following parameters were studied for all:

- Time of onset for blockade
- Total time for complete blockade
- IC_50_ for each drug.

### Percentage of block was obtained by:

twitch height in pretreated − twitch height in treatedtwitch height in pretreated

The inhibitory concentration 50% (IC_50_) values of each drug were calculated from the cumulative response curve (CRC), by regression analysis.

### Reversal of neuromuscular blockade by vecuronium and pantoprazole:

Effects of neostigmine (3.3 *μ*M), 3,4-diaminopyridine (0.25 mM), potassium chloride - KCl (6 mM), and calcium chloride - CaCl_2_ (10 mM) on both vecuronium (10 *μ*M)- and pantoprazole 4.0 mM)-induced paralysis were studied.

**Rabbit head drop method:** The procedure of the rabbit head drop method was followed as described by Verny.[23] The marginal ear vein was used for infusion of vecuronium (5 *μ*g/ml). The solution was infused from a 10 ml glass syringe fitted to a slow injector (INCO Biodevice, Ambala), which was adjusted for a speed of 1 inch every 10 minutes. After standardization of the technique, three groups of rabbits, consisting of six animals per group were used. In each group, time for the head drop (muscle supporting the head become sufficiently relaxed to prevent the head to be raised when the back is stimulated) after the infusion of vecuronium was recorded. Neostigmine (0.25 mg/kg) along with atropine (2 mg) was used to reverse the effect of vecuronium after the head drop.

**Table:** 

Group 1:	Used as control and received the infusion of vecuronium
Group 2:	(acute study group): received intravenous pantoprazole (1.9 mg/kg) 30 minutes before the infusion of vecuronium
Group 3:	(chronic study group): received pantoprazole (1.9 mg/kg) orally for 15 days before the infusion of vecuronium

**Statistical analysis:** Results were expressed as a mean ± SEM. The unpaired t-test was used to compare the difference in the cumulative response curves of pantoprazole in indirect and direct stimulation. One way ANOVA followed by Dunnett's multiple comparisons was used to analyze the different concentrations of pantoprazole on vecuronium-induced blockade. One way ANOVA was used to compare the three groups in the rabbit head drop method.

## Results

### Rat phrenic nerve hemidiaphragm preparation:

**Figure 1:**
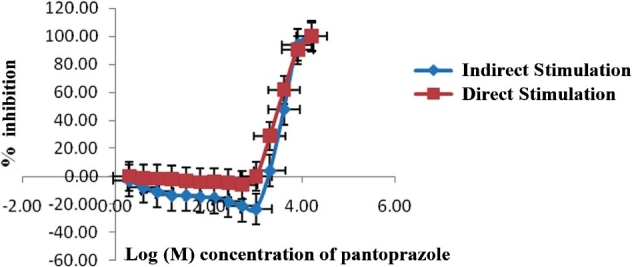
Effect of pantoprazole on the indirect and direct stimulation of rat phrenic nerve hemidiaphragm preparation Mean ± SEM has been shown (n = 6).

**Effect of pantoprazole on indirect and direct stimulation:** Pantoprazole (2 *μ*M to 16.0 mM) produced biphasic response on rat phrenic nerve hemidiaphragm preparation. There was an enhancement of basal contractile twitch response at the lower doses followed by blockade at higher doses with both indirect and direct stimulation [Figure 1]. There was no significant difference in the time of onset of the block (48.80 ± 1.56 and 44.44 ± 3.28 minutes), complete block (64.69 ± 1.56 and 64.36 ± 1.29 minutes), and IC_50_ (4.11 ± 0.23 and 3.89 ± 0.16 log dose) of pantoprazole paralysis in case of indirect and direct stimulations (Unpaired t-test: *P* > 0.05).

**Interaction of pantoprazole and vecuronium in the indirectly stimulated preparation:** Cumulative response curves of vecuronium (2 *μ*M to 16 mM) were taken in the absence and presence of three different concentrations of pantoprazole (32 *μ*M, 64 *μ*M, and 128 *μ*M) in indirectly stimulated preparations. Time of onset for block, time for complete block, and IC_50_ of vecuronium were compared between the control and pantoprazole groups. Quick skeletal muscle relaxation was achieved in the presence of pantoprazole, as shown by the significant reduction in the time for complete block. No significant difference was found for time of onset for block and IC_50_ of vecuronium in the presence of pantoprazole [Table 1].

**Table 1:** Effect of pantoprazole on vecuronium-induced blockade (Mean ± SEM, n = 6)

*Vecuronium*	*Time for onset of block (min)*	*Time for complete block (min)*	*IC_50_ (log dose)*
Control	6.71 ± 1.38	27.19 ± 0.84	1.08 ±0.15
Pantoprazole (32 μM)	6.46 ± 1.57	23.74 ± 1.16*	0.73 ± 0.17
Pantoprazole (64 μM)	7.59 ± 1.35	23.51 ± 0.84*	0.85 ± 0.14
Pantoprazole (128 μM)	10.17 ± 0.18	23.14 ± 0.91*	0.88 ± 0.15
One way ANOVA F (df)	1.89 (3, 20)	4.66 (3, 20)	2.89 (9,117)
*P*	> 0.05	< 0.05	> 0.05

* *P* < 0.05 compared to the corresponding control (Dunnett's multiple comparison Test). IC_50_-inhibitory concentration of vecuronium to produce 50% paralysis

**Effect of neostigmine, 3,4-diaminopyridine, potassium chloride, and calcium chloride on vecuronium- and pantoprazole-induced paralysis in the indirectly stimulated preparation:** Partial blockade of the basal contractile twitch responses of vecuronium (10 *μ*M) was reversed by neostigmine (3.3 *μ*M), potassium chloride (6 mM), and 3,4- diaminopyridine (0.25 mM), but not by calcium chloride (10 mM). Partial blockade of the basal contractile twitch responses of pantoprazole (4 mM) was not reversed by neostigmine (3.3 *μ*M), potassium chloride (6 mM), 3,4- diaminopyridine (0.25 mM), and calcium chloride (10 mM).

### Rabbit Head Drop Method

Time for the rabbit head drop after vecuronium infusion in the control, acute, and chronic study groups was: 4.74 ± 0.23, 1.99 ± 0.33, and 1.73 ± 0.13 minutes, respectively. Both acute and chronic administration of pantoprazole significantly reduced it, as compared to the control group (*P* < 0.001). However, the significant difference in time for head drop between acute and chronic study groups was statistically insignificant (Tukey's multiple comparison test).

## Discussion

There is evidence of the occurrence of interaction between PPIs and neuromuscular blocking drugs. Omeprazole intravenously, at therapeutic doses, alters neuromuscular function and enhances the action of atracurium and succinylcholine *in vivo* in rats.[20] Lansoprazole potentiates vecuronium-induced paralysis when used as a preanesthetic medication.[21]

In the present study, pantoprazole has shown dual action, with the initial potentiation of the basal contractile twitch responses at lower doses followed by paralysis at higher doses, in response to the indirect stimulation in the rat phrenic nerve hemidiaphragm preparation. It is similar to that produced by depolarizing neuromuscular blocking agents.[24] Our observations suggest that the direct action of pantoprazole on the skeletal muscle is responsible for diaphragm paralysis on both indirect and direct stimulation.[25] Absence of the reversal of the neuromuscular blockade of pantoprazole by neostigmine, and 3,4- diaminopyridine in the indirectly stimulated preparation suggests the noncholinergic or noncompetitive mechanism of the blockade. Similarly, the lack of effect of potassium chloride and calcium chloride on the reversal of the neuromuscular blockade of pantoprazole suggests that potassium and calcium antagonism may not be involved in its mechanism.[17] The potentiation of the basal contractile twitch responses in the preparation stimulated indirectly by lower concentrations of pantoprazole may be due to the weak anticholinesterase action of pantoprazole. The vecuronium-induced neuromuscular blockade was reversed by neostigmine, 3,4-diaminopyridine, and potassium chloride, but not by calcium chloride. Our observations on the reversal of vecuronium-induced neuromuscular blockade are in accordance with the previous studies.[17,26,27]

On rat phrenic nerve hemidiaphragm preparation there was no significant difference noted in the onset of blockade or the IC_50_ value of vecuronium, in the presence of pantoprazole. However, pantoprazole significantly reduces the time for a complete blockade in the cumulative response curve of vecuronium (*P* < 0.05), suggesting a potential interaction between them. In the rabbit head drop method, both acute and chronic administration of pantoprazole significantly reduced the time for the head drop by vecuronium infusion (*P* < 0.0001). This provides further evidence that pantoprazole produces a neuromuscular blockade. Whether pantoprazole metabolites potentiate the effect of vecuronium is not clear, but their contribution is probably minimal, as the potentiation is extremely rapid. It is possible that when neuromuscular transmission is impaired, such interactions may reach a clinical significance. Pantoprazole may impair the reversal of neuromuscular blocking drugs in some situations, such as, muscular dystrophies, patients with abnormal or low plasma cholinesterase activity, or those receiving drugs that depress neuromuscular transmission. It is suggested that one should carefully monitor the patients receiving pantoprazole as a treatment for peptic ulcer or preanesthetic medication, to prevent prolonged paralysis. However, the clinical significance of these results needs to be established further.
